# Repairing Vulcanite Plates

**Published:** 1884-08

**Authors:** George B. Snow

**Affiliations:** Buffalo, N. Y.


ARTICLE III.

### REPAIRING VULCANITE PLATES.

BY GEORGE B. SNOW, D. D. S., BUFFALO, N. Y.

The recent patent for a method of refitting vulcanite plates, which was published and commented on in the April *Advertiser*, has brought up a question as to what is extant in the text-books upon Mechanical Dentistry concerning this matter. The directions for repairing and refitting this style of work will be found very meagre, and it is believed that something can be written upon the subject which will be acceptable to at least the new beginners in dental practice.

It is not unusual to see vulcanite plates which have been cracked or broken, and repaired by what may be termed the "hole and plaster" system. Holes are drilled through the plate along the edges of the crack, and a new thickness of rubber superimposed upon a mass which possibly is already too thick for comfort or convenience; the old crack still remaining as a weak point to occasion further breakage. No advantage was taken of any possibility of union between the old and new material, the dentist having been obviously ignorant of the fact that perfect union can be obtained in such cases if the surfaces of contrast are freshly cut, absolutely clean, and properly roughened.

The fact that such bungling work is not uncommon must be the apology for a treatment of the subject more extensive in its scope than was originally intended, and a paper which cannot but be an only story to many readers.

The great point to be remembered in repairing or making any addition to a vulcanite plate is, that the new and old material will unite perfectly, and with such firm adhesion that the plate will be practically as good as new, if the surfaces of the old plate where union with the new material is desired are freshly filed, *absolutely clean*, properly roughened, and of sufficient area. To insure these results, wax should not be melted upon the surfaces of union in waxing up, and removal of the wax from the mould should be accomplished by means of instruments, and not by hot water, unless, possibly, for the removal of very small particles which cannot otherwise be got rid of. Any amount of the old material desired my be cut away, and its place supplied by new; and thus any change wished may be effected. In case of breakage or cracking, the plate should be cut away so that the old defects will be wholly obliterated and new material supplied.

As a first instance, suppose a partial lower plate, supplying the loss of the bicuspids and molars on both sides of the mouth, to be broken through the bar which extends from one side of the mouth to the other behind the incisors. The fracture is generally a clean one, resembling that of a glass or porcelain, and the two pieces may be brought into opposition with certainty. The dentist holding the parts together in exactly the right position, the assistant covers the lingual side of the plate at the point of fracture with a few drops of hot shellac from a shellac stick. A little cold water follows, and the two parts of the plate are firmly cemented together. A brace is now extended across from the molars on one side to those on the other, by laying a burnt match on the grinding surfaces of the respective teeth, and fastening both ends with a few drops of hot wax. By this means, sufficient strength is obtained to allow of the plate being safely handled. A piece of paper or sheet wax is cut to fit and reach across the lingual space at the lower edge of the plate and fastened therein with wax, a coat of shellac varnish is applied to the paper, the surface lathered with soap-suds, and rinsed, and a model run in the same manner as in filling an impression.

After this has hardened, the plate is removed from the model, which is then given a coating of liquid silex. This is always preferably done in repairing plates, at the time when the plate is first removed from the model. The bar may now be wholly cut away close to the body of the plate on either side by a jewellers' saw; the cut being made diagonally, so as to make what is termed a "scarf" joint. The surfaces should be further roughened by making a series of shallow parallel cuts across them with the saw, a thick separating file, or a thin wheel engine bur. The parts of the plate are placed upon the model, waxed up, and flasked; the model and buccal surfaces of the teeth being covered with plaster, and the parting made so that the plate will be retained upon the model, while the pieces of the bar can be readily removed. After the flask is opened, the pieces are removed, the usual gateways cut, and the packing, vulcanizing and finishing done as usual.

In the case of an entire lower set broken through the centre, it will be seen that the same directions will apply, excepting as to the amount of rubber to be cut away. A free cut should be made on the lingual side, extending through under the teeth, to and including the labial band; so that the broken surfaces will be entirely obliterated and at least one-eighth inch in width of new rubber supplied between the cut surfaces. An engine bur will do much of this work nicely, and a wheel bur is very convenient for the purpose of scoring the surface. The making a model, flasking, and packing will be done as before.

If one of the incisor blocks be broken, and needs replacement, a new one can be fitted after the model is obtained, and the remaining steps of the process followed as has been described.

Upper plates are sometimes cracked in the centre, the crack extending from under and between the incisor teeth backwards over the palate. This often happens from the amount of rubber just behind the incisors being insufficient. Is it not unusual to see it cut away at this point so that the pins are almost or quite exposed; the plate plate having its usual thickness at a very short distance behind the teeth. A much larger amount of material will be tolerated here than is usually employed, and often with benefit, not only to the strength of the plate, but to the articulation of the wearer. The curve of the surface of the plate should be made to resemble that of the palate before the removal of the teeth, and it will be found that the extra thickness that may extend for half an inch behind the teeth without annoyance to the patient.

A proper curvature to the surface of the plate, just behind the incisors, will do much to prevent the disagreeable whistling in making the *s* sound, and will assist in giving the correct enunciation to *sh*, *zh*, and other linguals.

If the cracked plate fits a flat mouth, a model can often be drawn from it as it is; but if the arch is high, and the gums projecting, it is better, after thoroughly cleansing and drying the plate, to finish the cracking by breaking the plate entirely in two. The two halves may now be fastened together by dropping shellac upon the lingual side, and a model secured, from which either half of the plate can be easily removed. The whole palatal portion of the plate can then be removed by a saw cut, leaving only a narrow margin on the lingual surface inside the teeth. The remainder of the surfaces of fracture are cut away as directed in case of the lower plate, the new surfaces roughened, the pieces of the old plate replaced upon the model (which has received its coating of liquid silex), waxed up, flasked, packed, and vulcanized, the teeth being retained upon the model as before described. The plate, when finished, will show the old rim and a margin of the old rubber inside the teeth.

It is sometimes desirable to change the substance of the plate entirely, as in case of supposed mercurial poisoning by red rubber; or at least to put what red rubber there may be about the plate entirely out of sight, and to reduce its quantity to a minimum. If this is to be done to the plate last under consideration, it should be prepared for flasking as described, excepting that the labial band should be cut away, and everything arranged so that the plate can be separated from the model when flasked. The parts cut away should, of course, be replaced by wax. The case is now set in the flask so as to leave the parting at the upper edges of the gums. The plaster is varnished and oiled, so that the appearance will now be precisely similar to a plate flasked so as to be retained upon the model. The ring of the flask is now put in place and filled, and the plaster allowed to harden.

When the flask is separated, the teeth will be found in its ring section. A few blows of the hammer will dislodge them, with the piece of plaster built against their labial surfaces. This is carefully broken away, in two pieces if possible which are preserved, and the teeth and rubber encasing them is left. The rubber is now filed away as much as practicable, leaving none of the old rubber in sight, and removing enough from the palatal surface to make a new fit to the model. The teeth and plaster are replaced in the flask, and the case is ready for packing and vulcanizing, and, when finished, none of the old rubber will be seen, and the plate will be practically as good as though the teeth had been removed from the old plate and reset.

It is sometimes difficult to prevent the rubber from showing at the joint between the incisors; great care should be exercised in bringing the sections together properly and in holding them in position while flasking. If there is room, a small wisp of loose cotton, not larger than a thread, may be tucked into the joint on its palatal side, the edges of the blocks being bevelled to admit of this being done.

It is evident that the change from red to black rubber just described can be made with a whole plate or a broken one indifferently. If a change of articulation and a new fit to the mouth is also desired, on account of shrinkage of the gums, the plate should be prepared so as to draw from the model, and a few small pieces of wax put in the palatal side to bear upon the alveolar ridge and give the right articulation by trial in the mouth: the centre of the plate being cut away to facilitate the fitting of the plate to the model. A fresh model of the mouth being secured from an impression, the plate is waxed on to it, the case is flasked with a false piece of plaster built against the labial sides of the teeth, as has been before described, and the plate afterwards removed and cut away as much as desired; a considerable amount being always taken from its palatal surface.

This process does all and more than is specified in the Hyatt patent, as it not only gives a new fit, but allows the material of the plate to be substantially changed. Holes and dovetails, it will be seen, are wholly unnecessary, and the fine serrated edge left by cross-cutting the surfaces of union will be found an excellent guide in scraping the plate to avoid overlaps. The use of shellac as a cement is strongly advised in repairing, as it is rigid and brittle when cold, and the broken parts, if once properly brought together, cannot get out of adjustment without at once attracting attention by the breakage of the cement. Wax does not answer the purpose nearly so well.

The amount of shrinkage in vulcanite from cooling after vulcanization is not so generally noticed and provided for as it should be. Plates composed of single teeth do not give trouble from this cause, but full plates, on which sections are mounted, are often very vexatious to the dentist from the change of shape they undergo from shrinkage.

The reason of this is, that the ends of the sections abutting from an arch of porcelain, which expands or contracts but slightly from changes of temperature. The rib of vulcanite immediately inside this arch, and in which the pins are embedded, forms a second arch closely attached by the pins to the first one. The plate is moulded to the model, and hardened at a temperature of about 320°, and is afterwards placed in the mouth, where the temperature is in the neighborhood of 90°. Under these circumstances the contraction of the rubber which ensues has the effect of lessening the radius of the arch, drawing the heels of the plate together, thus rendering it a little narrow to fit the mouth accurrately. This has the further effect of elevating the palatal portion of the plate, which, when tried in the mouth, will usually be found to rock slightly; often so much as to interfere with its fitting.

If the plate has been made upon a model taken from the mouth, the difficulty is overcome by warming the back part of its palatal portion, pressing it down slightly, and cooling it while the pressure is continued; the narrowing of the plate being too small in amount to be of itself objectionable.

This change can be accomplished with more certainty by making a small plaster cast of the palatal portion of the plate, placing upon the part where the change is desired a small piece of folded paper, and forcing the plate down upon it after its palatal portion has been warmed.

The shrinkage here alluded to becomes a more serious matter when the plate is revulcanized in the course of repairing it. It is flasked when the change in form by its shrinkage has already once manifested itself, and again heated at 320° ; and in cooling a second shringage takes place, it becomes still narrower, and its fit, already defective, is made perceptibly worse. It now becomes a matter of necessity to bring it back to its proper shape before it can be worn with comfort. To provide for this, a small dot should be made with a pointed instrument on each side of the plate immediately behind the molars, and a pair of dividers set to the distance between these points. After vulcanization, the dividers can be applied to the marks, and they will indicate the amount of shrinkage the plate has experienced. Let the plate now be warmed just behind the incisors, and in the mesial line, by repeated short puffs of a blow-pipe flame. This must be done carefully, and the heat not allowed to extend over an area much exceeding half an inch in diameter. When the rubber is sufficiently softened, the plate should be taken by the heels, a pull made upon it sufficiently forcible to expand the arch, and a stream of cold water applied. The dividers will at once show if the change made is sufficient.

When the plate is now tried in the mouth, it may be that the back edge will not touch the roof, and air will be admitted under the plate; in which case the back edge should be warmed and forced up to its proper position.

The same remarks apply to full lower plates as well, which often are found to have lost their fit in a measure, after having been revulcanized. The process above detailed will suffice to restore them to their former fit, and render them again comfortable to the wearer.—*Dental Advertiser.*

